# Performance measures to benchmark the grasping, manipulation, and assembly of deformable objects typical to manufacturing applications

**DOI:** 10.3389/frobt.2022.999348

**Published:** 2022-11-21

**Authors:** Kenneth Kimble, Justin Albrecht, Megan Zimmerman, Joe Falco

**Affiliations:** National Institute of Standards and Technology, Gaithersburg, MD, United States

**Keywords:** grasping, manipulation, assembly, performance benchmarks, deformable objects

## Abstract

The National Institute of Standards and Technology is developing performance tests and associated artifacts to benchmark research in the area of robotic assembly. Sets of components consistent with mechanical assemblies including screws, gears, electrical connectors, wires, and belts are configured for assembly or disassembly using a task board concept. Test protocols accompany the task boards and are designed to mimic low-volume, high-mixture assembly challenges typical to small and medium sized manufacturers. In addition to the typical rigid components found in assembled products, the task boards include many non-rigid component operations representative of wire harness and belt drive assemblies to support research in the area of grasping and manipulation of deformable objects, an area still considered to be an emerging research problem in robotics. A set of four primary task boards as well as competition task boards are presented as benchmarks along with scoring metrics and a method to compare robot system assembly times with human performance. Competitions are used to raise awareness to these benchmarks. Tools to progress and compare research are described along with emphasis placed on system competition-based solutions to grasp and manipulate deformable task board components.

## 1 Introduction

Assembly is one of the most complex operations in manufacturing with many fine robotic manipulation tasks still needing significant progression to achieve adoption within real-world robotic manufacturing applications. Adoption of these tasks requires implementations that minimize the use of specialized fixtures and single purpose end-effectors which significantly increase the time and cost to setup each new assembly process. Such strategies are paramount to the successful integration of robot systems that support the low-volume, high-mix manufacturing paradigm.

Assembly processes are primarily comprised of insertion and fastening operations such as threading, snap fitting, meshing, and routing. Rigid components used in these operations include screws, nuts, washers, gears, and electrical connectors. Additionally, assemblies include non-rigid components, also called deformable objects, such as belt drives and wires. Assembly processes invloving deformable objects such as wires, cables, and belts are in need of robotic solutions. The process of grasping and manipulating deformable objects for assembly is a difficult robotic task and an emerging research problem in robotics.

The National Institute of Standards and Technology (NIST) has developed a set of performance metrics, test methods, and assembly task boards (ATB) for benchmarking research and development efforts in robotic assembly (Kimble et al., 2020). Guidance for the use of these benchmarking tools, as well as the supporting artifact designs are available online at (Metrics and Methods, 2022). First introduced to the robotics community in 2017 in the form of competitions, these tools facilitate benchmarking among researchers to assess progress in the design and development of robotic assembly systems (Sun et al., 2021). The long-term goal of this work is the development of performance test methods for robotic assembly systems that will contribute to the generation of technical specifications for robot systems to ultimately aid in the selction of the best robotic system for an intended application space (Shneier et al., 2015).

This paper introduces the benchmarking concepts of NIST ATB test methods and artifacts with an emphasis on grasping and manipulating deformable objects. Section 2 provides some background on existing benchmarks to support grasping and manipulation and shows the need for deformable object assembly benchmarks that represent real-world robotic applications. Section 3 describes the NIST assembly task boards and associated test methods. Example primary task boards as well as competition task boards are described. Section 4 introduces the concepts of design-for-assembly used in the development of the task boards and provides examples of of time-based human performance baselines for comparison with the performances of automated assembly systems. Section 5 describes competitions that introduce these task boards to the research community and provides comparable performance results. A review of system performances during NIST hosted competitions reveals techniques that teams used to autonomously manipulate and assemble deformable object components. Section 6 introduces a new effort to provide a standardized object dataset to accompany the current ATB benchmarking tools. Finally, Section 7 summarizes these discussions and provides insight into ATB design improvements.

## 2 Towards deformable object benchmarks

The use of benchmarks to compare performance across robotic systems is gaining popularity within the robotics community. Unified collections of test metrics, methods, and object data sets using a common set of tasks allow researchers to assess incremental improvements between systems. Well defined and adopted benchmarks can be used to promote research in crucial problem spaces and foster competitive and novel solutions. The majority of benchmarks that support robotic grasping and manipulation are primarily focused on rigid objects and tasks associated with service robot applications.

Some examples of benchmarks focused on rigid body grasping and manipulation are now presented. GRASPA (Robot Arm graSping Performance BenchmArk) (Bottarel et al., 2020) is a benchmark to test the effectiveness of grasping pipelines on physical robot setups using a common set of rigid objects. Here, system assessment is designed to distinguish between failures caused by the testing platform and those introduced by the pipeline under test. The YCB (Yale, Carnegie Mellon, Berkeley) Object Dataset (Calli et al., 2015) is widely distributed and relies on the robotic community to develop accompanying test methods as benchmarking tools. The YCB organizers maintain a repository of benchmark protocols shared by users of the object set. The OCRTOC (Open Cloud Robot Table Organization Challenge) Benchmark (Liu et al., 2022) provides test setups for performing table organizations tasks with varying levels of difficulty utilizing remote hardware that is made available for researchers to test their algorithms. To support aerial robot research and development, (Suarez et al., 2020) evaluates aerial manipulation of rigid objects with regard to accuracy, execution time, manipulation capability, and impact response over a range of tasks including positioning, bi-manual grasping, load lifting, and contact force control.

Research in the area of robotic grasping and manipulation of deformable objects has gained ground in recent years and along with this research, supporting benchmarks are beginning to emerge. Examples of these benchmarks are sparse and those found are dissimilar to real world applications (Garcia-Camacho et al., 2020). developed a benchmark to evaluate bimanual robot tasks for grasping and manipulating textile objects of different sizes and types: spreading a tablecloth, folding a towel, and dressing, where each task is broken into sub-tasks for incremental evaluation of varying levels of difficulty (Garcia-Camacho et al., 2022). propose a cloth object set to support the robotics cloth manipulation community. This set of household cloth objects is being distributed in hopes to design common benchmarks using a community driven approach. Soft Gym (Lin et al., 2020) is a simulation based deformable object benchmarking tool that goes beyond textile objects and includes linear deformable objects such as rope to assess robotic grasping and manipulation solutions (Chatzilygeroudis et al., 2020). propose benchmarks to assimilate two manufacturing operations involving deformable parts, assembly of a watch plate, and a belt drive. Watch parts are replicated to be larger though 3D printer replication. A rubber band and 3D printed pegs are used in place of an actual belt drive. It is evident from the literature that more realistic object sets are needed to represent specific application spaces.

Linear deformable objects such as wires and bundles of wires (cables) are the prevalent flexible components used in manufacturing assembly applications. Additionally, belts used in mechanical drives to transfer rotary motion can often be found in assemblies. In the case of wire harnessing, fixtures such as cylindrical pegs, clips, and tubes are used to route wires/cables to form the final wire harness assembly. This process to construct the wire harness accounts for 70% of the total production time where up to 90% of this process is manual (Nguyen et al., 2021). In contrast, preparatory stages where wires with different cross-sections are cut into predefined lengths, stripped, and crimped are highly automated, and manual work is only required for quality assurance, machine-setup, and maintenance. The last process is to assemble the final wire harness.

With respect to the grasping and manipulation of deformable objects for manufacturing assembly applications, emerging research areas include gripper and manipulator design, sensing, modeling, planning, and control (Zhu et al., 2022). Because this is a complex, highly manual assembly operation, there are several instances of research efforts which attempt to solve this robotic automation problem. Some of these solutions use collaborative robot systems to enable the tasks to be divided between robots and humans (Heisler et al., 2021). Jiang et al. investigates the design of a multi-robot system for installing the completed wire harness into the body of an automobile (Jiang et al., 2010). The ARM (Advanced Robotics for Manufacturing) Institute, a Manufacturing Innovation Institute (MII) funded by the United States Office of the Secretary of Defense and part of the manufacturing USA network has identified several key focus areas and funded projects in the areas of flexible material handling and assembly of composites, textiles, and wire harnessing (ARM (2022)).

Research in this area would benefit from a set of unified test methods and artifacts to benchmark research progress as well as to compare different approaches to solving the problem. Ideally, these benchmarks could also mimic the problem space that small and medium sized manufactures who most often produce in batches with product variation from batch to batch. To support production in such an environment where robot expertise is often limited, and cost is always a factor, robot systems must be easy to deploy and reconfigure with minimal retooling. NIST assembly task boards introduce linear deformable objects typical to the manufacturing assembly application space in the form of wires and belts and associated benchmarks that replicate real world applications. The majority of the design choices for these deformable object based benchmarks and associated task board components were derived from reviews of typical mechanical assembly designs as well as by robotic assembly research focus areas as identified by the ARM institute.

## 3 NIST assembly task boards

**FIGURE 1:**
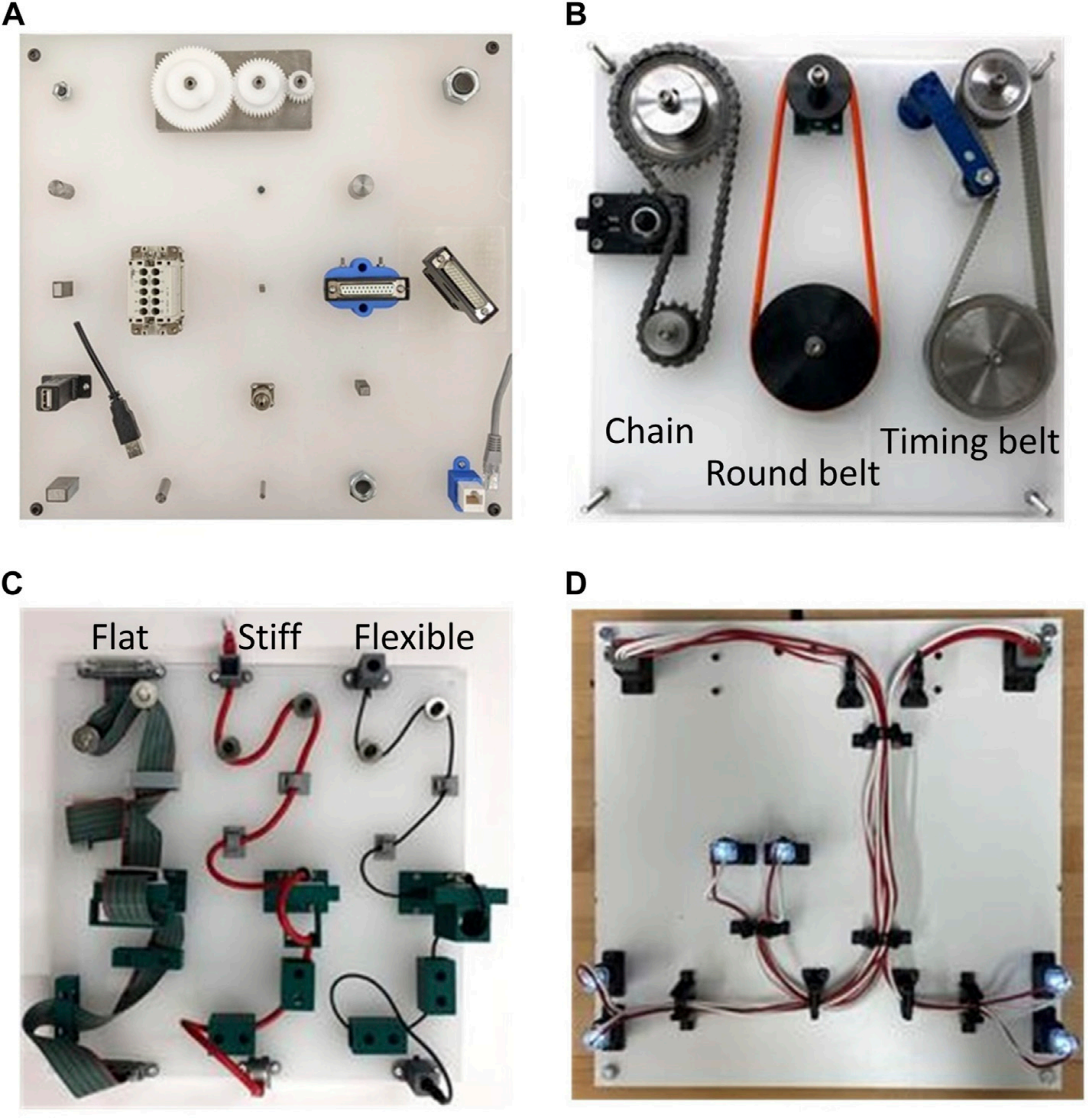
NIST Assembly Task Boards (ATBs): **(A)** ATB1 - peg insertions, gear meshing, electrical connector insertions, nut threading; **(B)** ATB2 - alignment and insertion of collars and pulleys, handling flexible parts, meshing/threading belts, actuating tensioners, and threading bolts; **(C)** ATB3 - tracking, placement, weaving, and manipulation of loose cables, handling flexible parts, and inserting ends into various connectors; **(D)** ATB4 - placement, weaving, and manipulation of wires, pin insertions, bundling of wires, and connecting/disconnecting harness from connectors.

Motivation for the creation of the task boards comes from the need for a unified set of benchmarks that allow researcher to adopt a known procedure and compare results across a larger audience in a reliable and repeatable manner. The ATBs are designed to incorporate standard off-the-shelf components of varying sizes that are representative of components typical to manufacturing applications (Kimble et al., 2020). Each of the four ATBs presented below represents a subset of typical manufacturing assembly tasks. The ATBs provide a means of benchmarking the capabilities of a robot system by establishing a procedure and scoring metric for each task associated with the board. The task boards along with a description of each are provided in Figure 1.

### 3.1 Protocols for using the task boards

The procedure for using any of the NIST assembly task boards is broken down into two sub-tasks, disassembly and assembly. During disassembly a fully assembled board is placed on a surface and an empty bin is placed within the work volume of the robot system. The goal for the robot system is to remove all components from the board and place them in the bin. The robot system begins removing each part from the task board, one part at a time, until all parts have been removed. The finish time is recorded.

To start the assembly process, a fully disassembled board is placed on a surface alongside a kit-mat and any feeder or binning mechanisms necessary for assembly. Each board has a kit-mat specifically designed for its assembly that provides the specific location and orientation of a start position for each part. The robot system begins grasping each part from the kit-mat and assembling it into the board. Each part is assembled and scored one at a time until the entire board has been assembled. A finish time is recorded. The protocol steps for disassembly and assembly are shown in Table 1.

**TABLE 1:** Disassembly and assembly protocols.

	Disassembly	Assembly
1	Place the task board within the robot system work volume (task board position and part locations are fixed or random per system capabilities).	Place the task board within the robot system work volume (task board position and part locations are fixed or random per system capabilities).
2	Place the container to receive disassembled parts within the robot system work volume (container position is fixed or random per system capabilities).	Place the kit of parts to be assembled within the robot system work volume (kit position and part locations are fixed or random per system capabilities).
3	Initialize timing, recording the start time *T*_*start*_.	Initialize timing, recording the start time *T*_*start*_.
4	If used, perform manual programming.	If used, perform manual programming.
5	Start autonomous operation of the robot system.	Start autonomous operation of the robot system.
6	The robot system disassembles a part from the task board.	The robot system grasps a part from the kit layout.
7	The robot system places the removed part into the associated container.	The robot system assembles the part onto the task board.
8	Repeat steps 6 and 7 for all parts in task board.	Repeat steps 6 and 7 for all parts in the kit.
9	Record the finish time *T*_*finish*_.	Record the finish time *T*_*finish*_.
10	Repeat this protocol for the desired number of trials of the task board under test.	Repeat this protocol for the desired number of trials of the task board under test.

**FIGURE 2:**
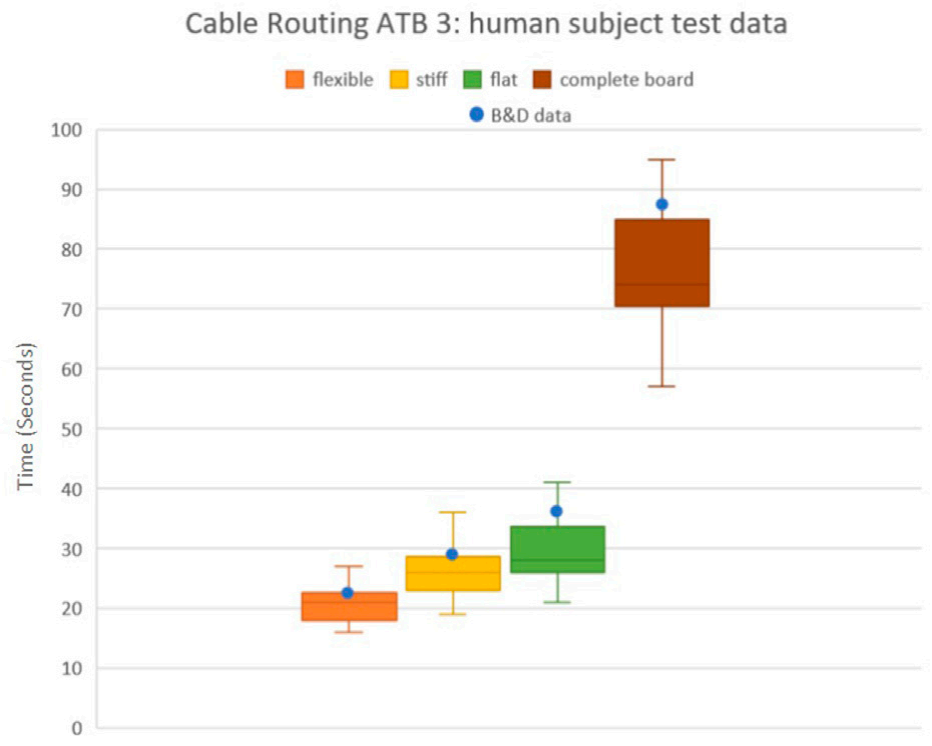
Calculated experimental time based on Boothroyd tables compared against the local pilot data of three human subject participants, ten trials each for assembly of ATB 3. Data is shown per wire type as well as entire task board operation.

The protocol and task board benchmarks can be used test a robot system’s ability to recognize, grasp, and assemble/disassemble small parts. The option to randomize task board and kit layout placement provides localization uncertainties that must be resolved by the system under test. The boards can be used to evaluate incremental system design improvements as well as compare research results across the robotics research community. Ideally, good system designs include the use of perception such as machine vision and force sensing. Since in manufacturing design, part data is readily available as Computer-Aided Design (CAD) data, it can be leveraged to solve the autonomy problem. Grasping type end-effector designs can also be evaluated using these benchmarks Figure 2.

### 3.2 Adoption of the assembly task boards

NIST ATB 1 was established in 2017 and has been utilized in many research efforts since its creation. Some of the most notable implementations of the board are listed here. Wenzhao et al. used the protocols established by the NIST ATB as a means of testing off-the-shelf robotic solutions. They also used ATB 1 as the benchmark for their performance (Lian et al., 2021). Thoo et al. (2021) used ATB 1 to compare offline robot programming to online using augmented reality. Narang et al. (2022) used the rigid bodies of ATB 1 to establish a robot assembly test suite. Luo et al. (2021) perform a thorough comparison of Deep reinforcement learning(DRL) from demonstration, against results of a professional industrial integrator on the established NIST benchmarks. NIST ATB 2 saw its early use as the inspiration for The World Robot Summit assembly challenge, (Yokokohji et al. 2022) describes the details and results of this competition.

## 4 Contrasting time-based human performance

The design of each ATB utilizes reasoning put forth by design for assembly (DFA) studies (Boothroyd et al. 2011). The studies have identified and tabulated various important factors based on manual human performance in assembly based tasks. For instance, how the size and symmetry of parts, tool usage, fixturing, mechanical resistance, mechanical fastening processes, visual occlusion, and physical obstruction all influence time-based human performance. Each task presented by Boothroyd is broken into its most elemental state, e.g., the time it takes to grasp an object of a particular size is separated from the time it takes to insert the object. Each of these recorded times notes the mechanical differences in assembly such as edges with chamfers or ease of grasp. Boothroyd seeks to encourage good mechanical design to decrease production time in assembly operations.

The particular details of each part such as shape, size, material, stiffness *etc.* Were chosen for each ATB based on the tables presented by Boothroyd. The large amount of data made it unrealistic to create a task board presenting all the possibilities of parts so a select few were chosen. The pegs for ATB 1 were chosen to span the limits of the recorded data making sure to select a peg from each possible design parameter (Boothroyd et al., 2011, p. 83). Given the limited benchmarking efforts prior to this work it was important to make a decision for each part so that future users of the ATB are performing tests with the same repeatable parts.

The results of the studies present enough time-based data that a theoretical model of nearly any assembly operation time could be predicted by adding together the time for each individual operation of the assembly. This paper presents a means of utilizing this data to create the “calculated experimental time” for task board 3. This same process could be applied for each of the four task boards, but given the scope of this paper being on deformable objects, only the cable routing tasks were chosen for the experiment.

Boothroyd et al. present example tables with calculated times for wire dressing based on the number of wires and whether or not access is restricted (e.g. a 1-wire flexible cable might take 6.3 s to dress). Another example presents assembly insertion times for cable ends with assembly parameters for orientation required, alignment features, and resistance to insertion (e.g. a single insertion of an Ethernet cable would take 2.5 s according to this table). Boothroyd breaks wire-types into three categories, namely a single flexible wire, a stiff multi-wire, and a flat cable. Furthermore, Boothroyd adjusts handling time based on the length of the wire (Boothroyd et al., 2011, p. 178).

### 4.1 Calculated experimental data

The wires/cables for Task Board 3 were chosen based on widely used commercially available materials that fit the categories determined by Boothroyd, namely an audio jack cable, an ethernet cable, and serial advances technology attachment (SATA) flat cable. The ATB 3 assembly, shown in Figure 1C, requires the three wires/cables be manipulated through various obstacles. Each wire is scored based on the completion of the sub-tasks along the length of the board.

Based on the various tabulated results presented by Boothroyd et al. an experimental calculation was generated to estimate the time it might take a user to complete an assembly of ATB 3. Table 2 shows the resulting experimental times for ATB 3. Certain assumptions had to be made regarding the details of the experimental times. Boothroyd did not provide a complete description of the objects or environment of the experiments such as the material of the items or the geometry of the work space. This is likely due to these factors not significantly affecting the handling time.

**TABLE 2:** Estimated time for assembling wire from Boothroyd and average time for human subjects to assemble the entire ATB 3 averaged across three participants, 10 trials each. All units are in seconds.

Wire type	Length (ft)	Handling	Routing	Dressing	Insertion	Boothroyd total	Subject data
Flexible wire	3.6	5.58	7.08	7.72	1.9	22.28	20.94
Stiff wire	3.4	8.78	10.28	7.48	2.2	28.74	26.97
Flat cable	3.3	9.98	12.14	11.38	2.5	36	30.47
Complete Task Board						87.02	78.39

### 4.2 Local pilot data

A series of experiments were performed by three human subjects initially unfamiliar with the tasks associated with the board. This test was done to validate the calculated experimental data. Test subjects were tasked with the assembly and disassembly of ATB 3 using both hands. Subjects began by routing the thin audio cable and inserting its connector at the end of the board. The subject then moved to the thick ethernet cable. Lastly, the subject assembled the flat SATA cable. The overall time was recorded for assembly, then subjects were asked to disassemble the board in reverse order. The subjects were purposefully not given specific instructions on the best practice or strategy. Over a series of 10 trials each subject’s average performance was recorded for each wire as well as the board as a whole. The resulting data presented in contrast to the calculated “Boothroyd et al. 2011 Total” is presented in Table 2 and Figure 2. The calculated results match closely with the local pilot data presented below. Test subjects’ averages for the flexible wire were within 93% of the calculated values. The subjects’ averages for the stiff wire were within 93% of the calculated values. The subjects averages for the flat cable were within 84% of the calculated values. End-users of ATB 3 might use this data as a goal for completion time using their robot system for handling flexible parts. This study was limited to colleagues currently working on the project as it did not require any additional work or assistance to perform. The study was used for collecting subject data in an effort to provide expected handling and assembly times for known flexible parts. User feedback regarding the protocol or effectiveness of the experiment was not collected.

## 5 Competition solutions for handling deformable objects

**FIGURE 3:**
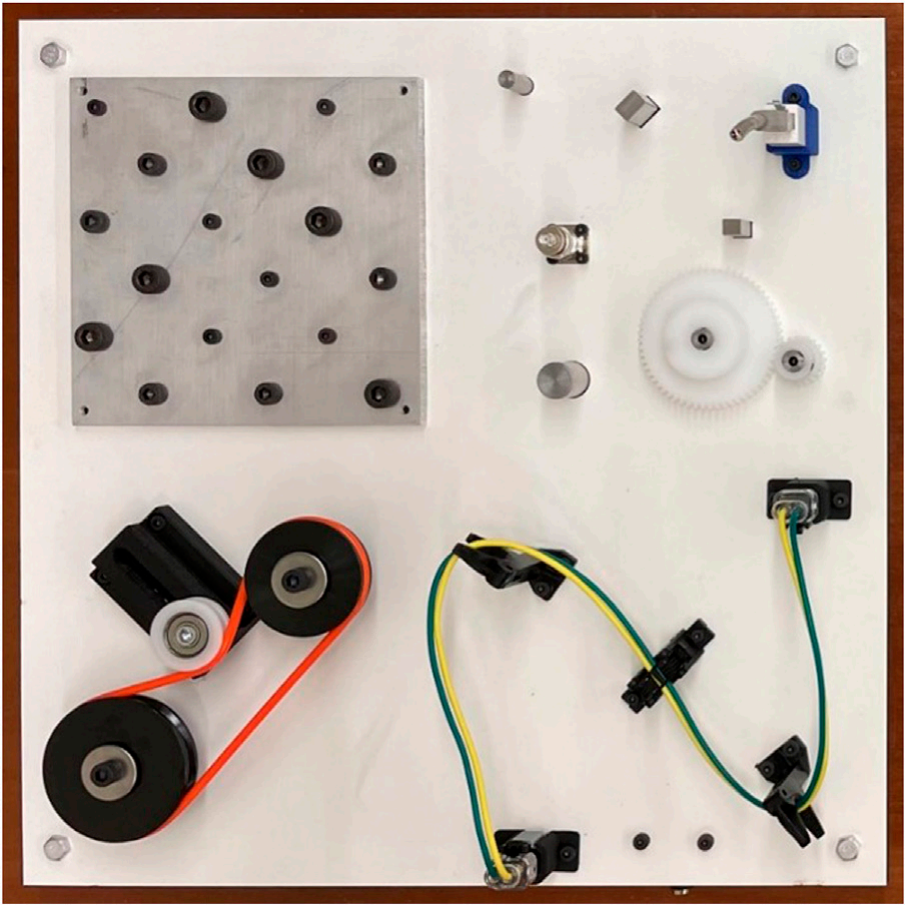
ICRA2022 Grasping and Manipulation Competition: Manufacturing track task board with four quadrants of the following themes: (top-left) Threaded Fasteners, (top-right) Insertions, (bottom-left) Belt Drive, (bottom-right) Wire Harness/Routing.

The Robotic Grasping and Manipulation Competition (RGMC): Manufacturing Track has been used over the past several years as a mechanism to introduce these benchmarking concepts to researchers as well as to provide feedback on areas for improvement. (ICRA (2022); IROS (2020); IROS (2019); IROS (2017)). A new task board is designed for each competition that presents a collection of tasks from each of the primary NIST ATBs. The most recent competition task board from the RGMC held at ICRA 2022 is shown in Figure 3. Tasks are divided into quadrants that represent four themes; fasteners, insertions, belt drive, and wire harness/routing to provide best-in-class recognition for each area in addition to a total score. The tasks for the competition boards are subject to change each year eventually allowing every aspect of the NIST ATB to be presented. Teams were given a time limit to disassemble the competition board and a separate time limit to assemble the board. The time limitations are enforced to discourage the use of teach-style programming. A randomly placed task board with unknown part positions and associated computer-aided design (CAD) files are presented at time zero, requiring teams to use a robot system that can be rapidly reconfigured. This scenario is representative of the low-volume, high-mixture manufacturing paradigm and the availability of CAD data typically accompanying assembly designs.

**FIGURE 4:**
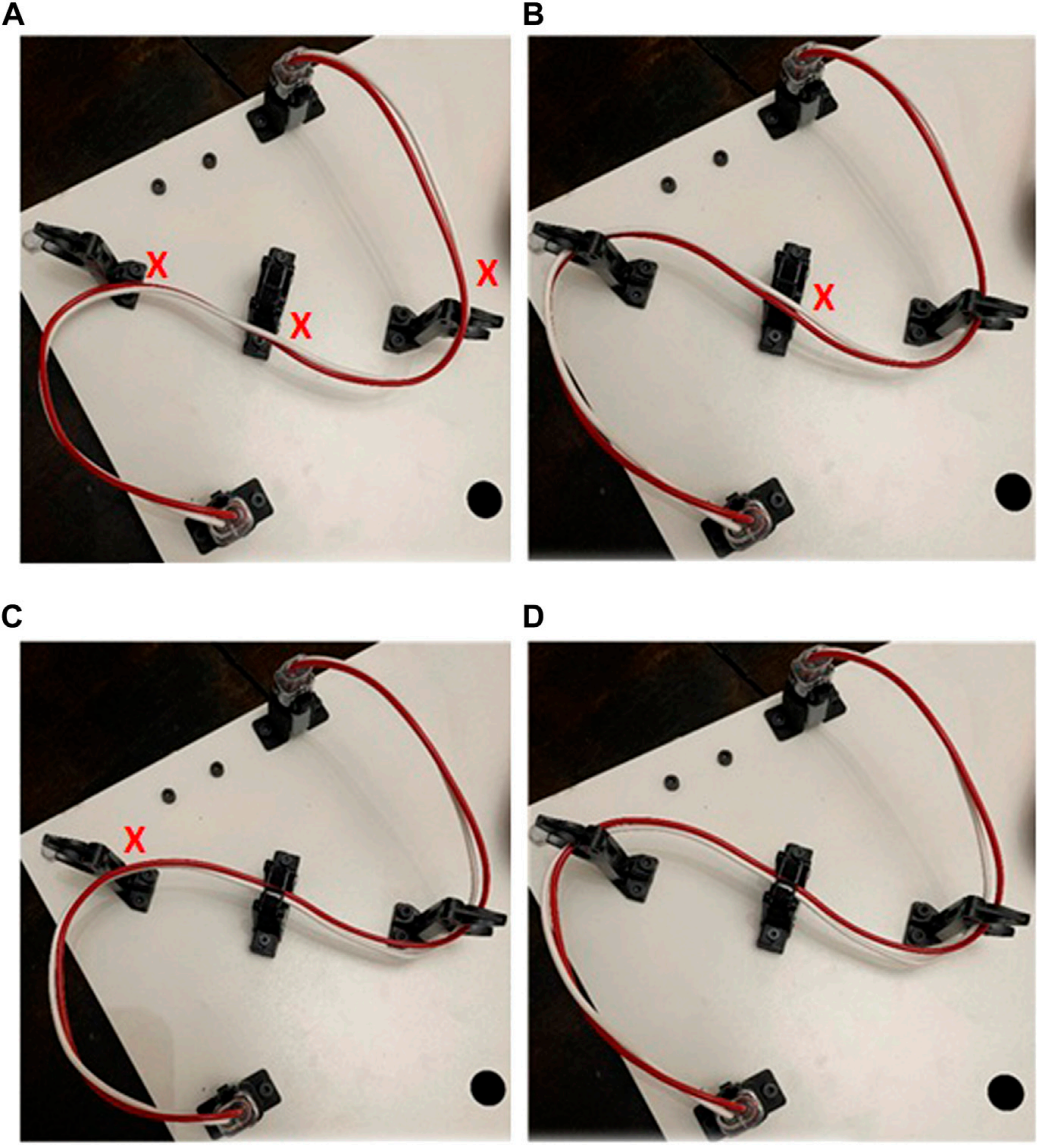
Scoring for assembly of wire harness equates to 6 points per wire insertion into contact retainer: **(A)** 0 points, no wires routed through any retainers: **(B)** 24 points, two wires each routed through two retainers; **(C)** 24 points, four routed through retainers and; **(D)** 36 points, two wires each routed through three retainers.

**FIGURE 5:**
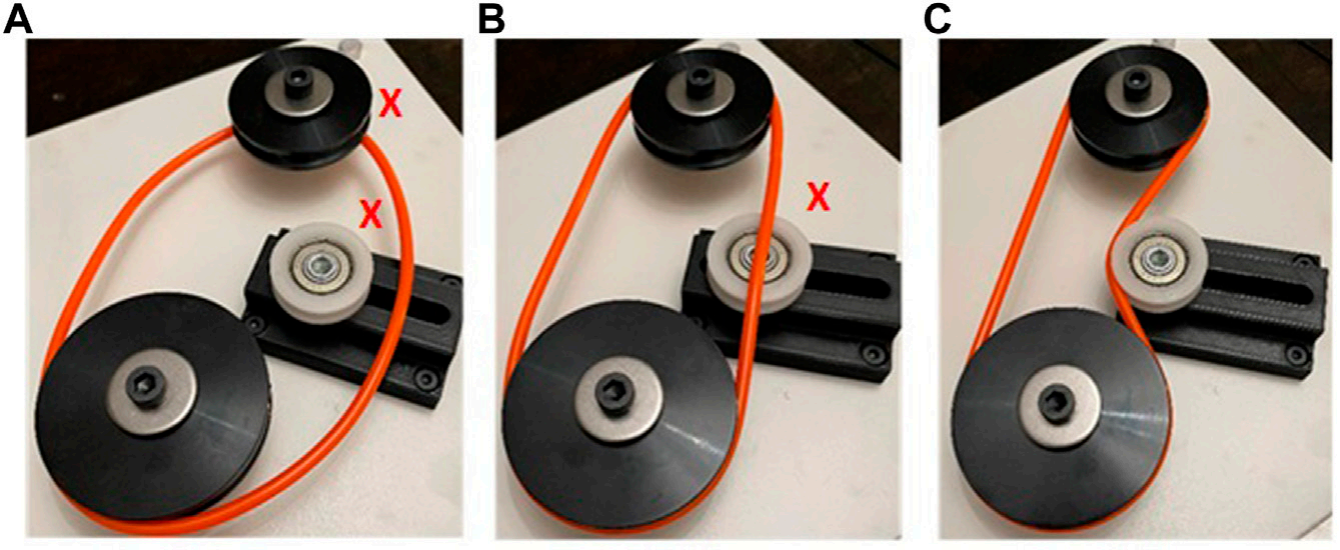
Scoring for assembly of the belt drive equates to 14 points per belt threading operation and 14 points for tensioning: **(A)** 14 points, threading one pulley; **(B)** 28 points, threading two pulleys; **(C)** 42 points, threading two pulleys and tensioning belt.

Scores for the competition are assessed on a per part basis where each part receives points based on the completion of its sub-tasks. Separating sub-tasks into partial points in this manner mimics the data presented by Boothroyd and documents the specific assembly processes that are problematic for a robot system. An example of a partial score for the wire harness task is shown in Figure 4, and for the belt drive in Figure 5. The performance and feedback from teams to date indicates that flexible parts are clearly an area where research is needed as teams will often accept partial points for an incomplete task or opt not to try any of the deformable object assembly tasks. With this in mind, subsequent competitions will aim to create a lower barrier of entry for teams looking to score points on the deformable object sections of the board, the goal being to encourage research to address these specific deformable object assembly tasks.

The competitions created a challenge for teams that required unique solutions to handling deformabe objects. Understanding the solutions that teams are using helps drive the future competition designs to ensure that they are always progressing the technology. The hope is that these competitions could be used to drive real solutions in industry and further increase the reach and impact of the NIST established performance measures and benchmarks.

Various solutions were implemented by teams attempting to assemble the flexible elements of the task boards, namely the belt and universal serial bus (USB) cable. Analysis of competition videos revealed several strategies, the first of which localizes the deformable object to a known position in order to assemble the object. For the belt, competitors with one robot arm typically attempted to catch the object on one of the pulleys to use as an anchor, then pull until a desired force was reached. By stretching the belt to a given force the competitor could more easily estimate the actual position of the belt. In addition if the robot moved to a known position and no force was felt at the end-effector it could be determined that the belt was not properly seated on the pulley. For competitors that used two arms the belt was grasped on opposite ends by each arm then stretched to a known position using position and force feedback. Gripper fingers were designed to be narrow in order to keep the belt close to the second pulley during the seating process which also eliminated over stretching the belt.

**FIGURE 6:**
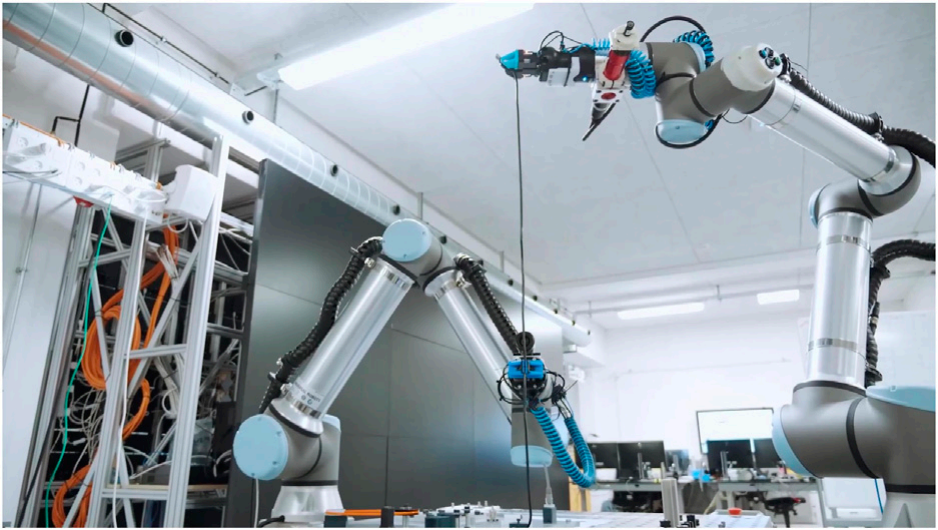
A Team utilizing two robot arms to solve the task board by extending the USB cable to its full length prior to assembly.

When attempting the USB cable assembly the competitor with two robot arms would anchor one end at table with the first arm then stretch the cable to its full length vertically with the other arm, see Figure 6. When a desired force was reached the competitor knew that the cable was fully extended and therefore knew the starting configuration of the cable. Using two arms to reach a desired initial position of the deformable object appears to be more reliable and faster than the single arm approach.

**FIGURE 7:**
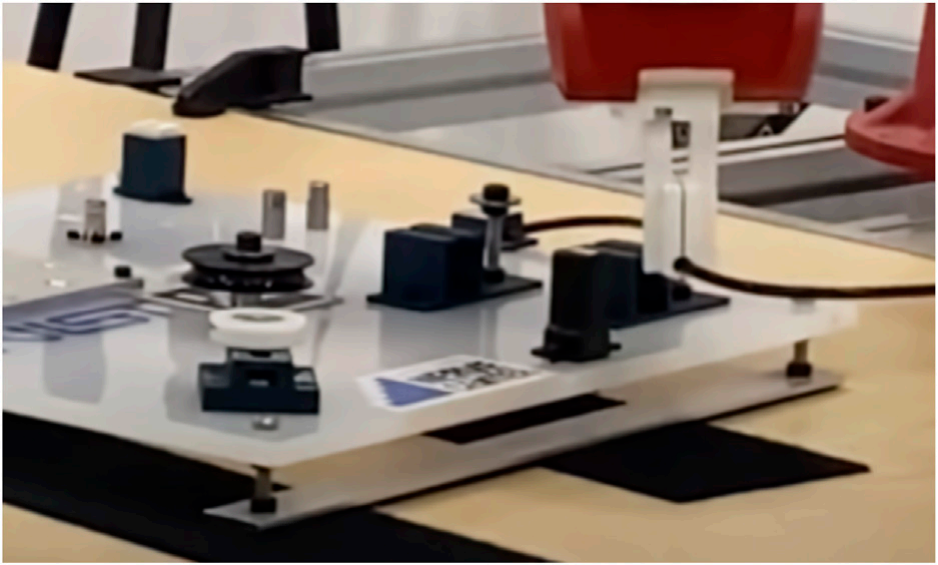
A Team utilizing a specialized gripper for cable assembly that allows the cord to be grasped tightly at certain points while also allowing for the position along the length of the cable to be adjusted without needing to re-grip.

At least one competitor noted using a compliance controller for handling the assembly of task board and specifically mentioned that the stiffness and damping parameters for the controller were modified during assembly of the cable and belt (Gorjup et al. 2021). Another strategy utilized by a few competitors is the use of a specialized gripper suited to cable assembly. The gripper works in two different modes. One where the cable is sufficiently pinched by the gripper so that it can be moved without slippage, and another mode where the robot arm can move while slipping to grasp the cable at another point along its length. During the motion along its length, the circular shape of the gripper end would prevent the object from being fully detached from the robot. This was most effective in the assembly of the USB cable where the robot could pinch the cable with enough force to move certain sections of the cable into the management slots then move along the length of the cable to begin working on another slot without having to re-grasp the object, see Figure 7.

## 6 Manufacturing Objects for Assembly Dataset

Methods of recognition and representation of deformable objects include the use of deep learning for processing sensory data. In the case of linear deformable objects found in manufacturing applications, there are typically connectors at the ends of the wires/cables. These semi-deformable linear objects (SDLO) provide a known part that can used for training a neural network (Zhou et al. 2020). used vision based learning techniques to solve for the position of the rigid body at the end of the SDLO with an average error of 0.316 mm and 0.211°. They were able to use this estimation to reliably grab the connector and manipulate it into a receptacle using a dual arm robot. A dataset of 2D and 3D images of manufacturing based parts like the connectors in SDLOs, would greatly benefit deformable grasping, manipulation, and assembly research.

Vision based models are used in tasks like rope manipulation and the folding of cloth materials and similarly can support wire manipulation and harness manipulation tasks. Data collection for such materials can include static image information and video demonstrations. In such systems, visual occlusions are a concern, including self occlusion (Zhu et al. 2020). proposes that these occlusions can be compensated for by using vision data collected with different perspectives.

A Manufacturing Objects for Assembly Dataset (MOAD), consisting of of 2D and 3D visual sensor data, will be developed at NIST to support robot system solutions utilizing NIST task boards. The YCB database provides high-resolution red green blue depth (RGBD) scans, physical properties, and geometric models of the objects for easy incorporation into manipulation and planning software platforms (Calli et al. 2015). The YCB dataset does not support multiple representations of the deformable objects with different self occlusions. The YCB dataset does include object mass properties and dimensions which are properties inherent in the ATB component manufacturing CAD data.

**FIGURE 8:**
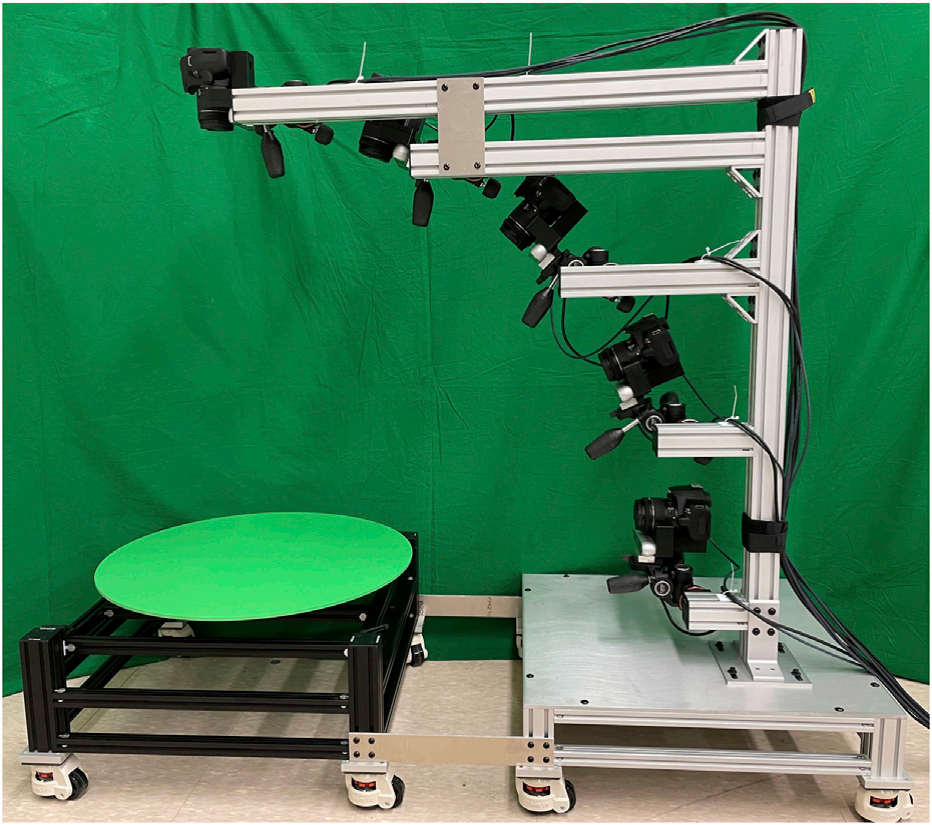
MOAD Apparatus: used for gathering object data.

Figure 8 Shows the MOAD apparatus which acts as a reliable and repeatable means of collecting image data from multiple angles of a part. The apparatus presents a series of high resolution and Laser Imaging, Detection, And Ranging (LIDAR) cameras in an array from 0 to 90°, incremented at 22.5° intervals. Each of the cameras point to a central point on a motorized platform where the object is centered. The platform is programmed to rotate in 5° steps. Images are captured by each camera at every step of the rotation creating a large dataset of images for any object of interest.

Representations of deformable objects in MOAD will include examples of the objects with multiple orientations and self occlusions. Video samples demonstrating the behavior of the deformable object as it is manipulated through its respective assembly are also pertinent. In addition to the static data of objects, there are plans to collect data of the flexible materials being manipulated standalone as well as being manipulated within the ATB by human agents, robot agents, and human robot teams, including vision data from a variety of perspective angles. Such data can be used to enable sim-to-real reinforcement learning solutions, and provide models with deformation information from a variety of angles (Matas et al. 2018). It is conceivable that the MOAD data set will reduce the barrier of entry for the usage of the NIST ATBs and enable the development of novel solutions for robot manipulation for flexible material assemblies.

## 7 Conclusion and future efforts

This paper provided an overview of a set of assembly task boards and the associated test protocols for benchmarking research in grasping, manipulation, and the assembly of typical manufacturing components. The protocols presented are designed to emphasize the challenges found in low-volume, high-mixture manufacturing applications typical to small and medium sized manufacturers. The ATBs are gaining acceptance as a means of benchmarking and comparing research within the robotics community and ATBs with deformable objects are slowly being introduced through the primary ATBs as well as through competition ATBs used in the RGMC Manufacturing Track.

A more in-depth study to be performed in the future will determine the calculated experimental data for all task boards and a larger sample size of human test subjects will be used. A process for determining the calculated experimental data using Boothroyd’s work will be laid out so that future users of NIST ATB are provided with a baseline of assembly time for comparing time-based human data to their robot system.

**FIGURE 9:**
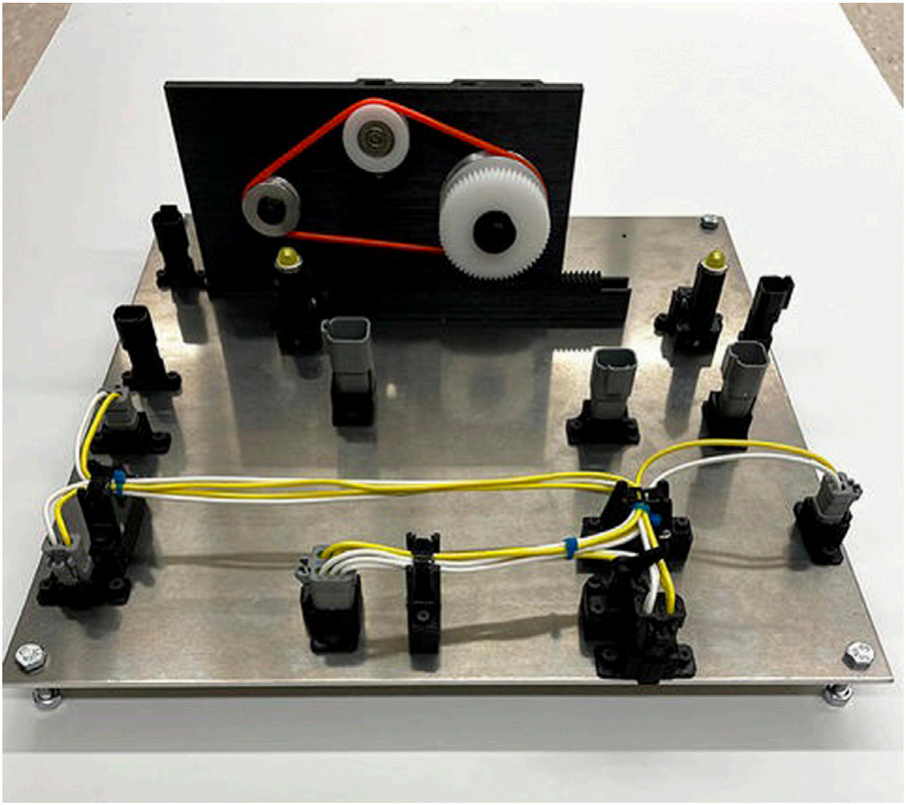
A future ATB design that includes building a wire harness and assembling the wire harness to provide power to a belt drive unit.

A future task board prototype is shown in Figure 9. In addition to the assembly of a wire harness, this ATB also includes the task of assembling the completed wire harness to provide power to a motor driven belt drive unit also assembled autonomously by the robot system under test. If the belt drive is properly assembled and the wire harness is built and assembled correctly, the belt drive can be operated to provide linear motion to a rack and pinion gear set for cyclic motion between two limit switches where an light emitting diode (LED) indicator shows when the limit switch is engaged.

The next steps for Manufacturing Objects for Assembly Dataset is to use the developed apparatus and recording technique to generate 2D and 3D sensor data of each part, sub-assembly, and completed assembly. In addition to this data, video footage of the assemblies being performed, CAD models of each part, and additional pertinent metadata regarding lighting environments and calibration information will also be provided. Finally, a detailed set of instructions for replication of the data collection rig and process will be documented and made available to researchers.

NIST’s work for ATB 1–4 has been recorded but this work is ongoing and likely to add more boards as the technology improves and industry needs change (Kimble et al. 2020; Metrics and Methods 2022). This work is also being considered for standardization under ASTM International Committee F45 on Robotics, Automation, and Autonomous Systems, Subgroup F45.05 Grasping and Manipulation.

## Data Availability

The original contributions presented in the study are included in the article/Supplementary Material, further inquiries can be directed to the corresponding author.
